# Supplementary material for: The WHHERE coactivator complex is required for retinoic acid-dependent regulation of embryonic symmetry
Source: Nat Commun. 2017 Sep 28;8:728. doi: 10.1038/s41467-017-00593-6 (PMC5620087; doi:10.1038/s41467-017-00593-6)
Supplement: Supplementary file 2 — Supplementary Information [file 41467_2017_593_MOESM2_ESM.pdf]

File Name: Peer Review File

File Name: Supplementary Information

Description: Supplementary Figures, Supplementary Table, Supplementary Note, Supplementary References.

File Name: Supplementary Data 1

Description: List of Rere-asssociated proteins identified by MudPIT.

Supplementary Information

Supplementary Figure 1

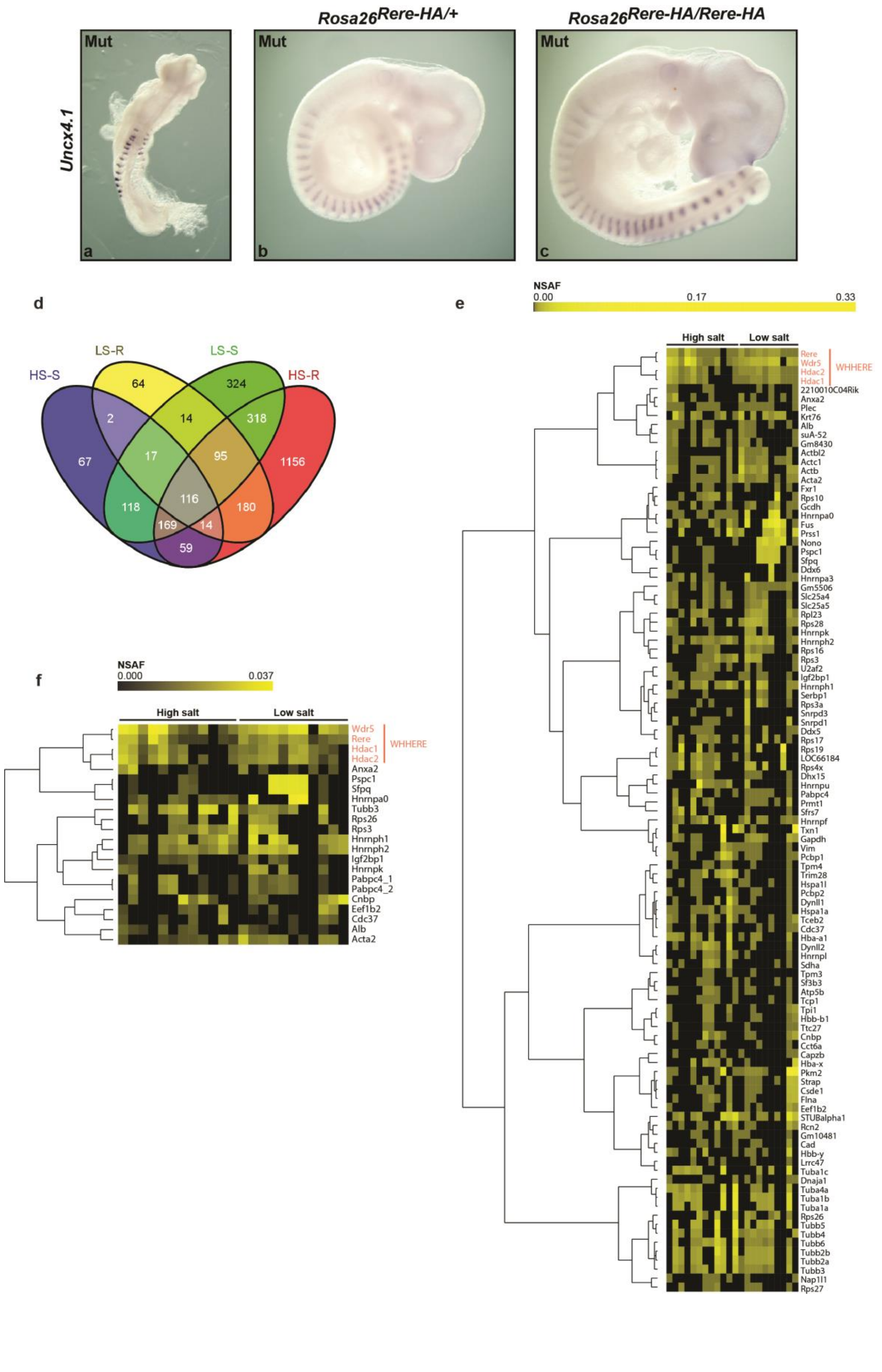

Supplementary Figure 1 (related to Figure 1) Identification of Rere-associated proteins using multidimensional protein identification technology (MudPIT).

(**a**-**c**) Rescue of *Rere*$^{om/om}$ embryos by ubiquitous expression of Rere-HA from the *Rosa26* locus. *In situ* hybridization with *Uncx4.1* in E8.75 *Rere*$^{om/om}$ (**a**) (dorsal view), E9.5 *Rere*$^{om/om}$*;Rosa26*$^{Rere\text{-}HA/+}$ (**b**) and E10 *Rere*$^{om/om}$*;Rosa26*$^{Rere\text{-}HA/Rere\text{-}HA}$ (**c**) mouse embryos (lateral views). *Rere*$^{om/om}$ mutant embryos (Mut).

(**d**) Venn diagram comparing the lists of proteins identified after Rere-HA immunoprecipitation in the four different conditions. HS-S: High Salt and Sigma HA beads, LS-R: Low Salt and Roche HA beads, LS-S: Low Salt and Sigma HA beads, HS-R: High Salt and Roche HA beads. The intersection of all four conditions identified 116 distinct entries that correspond to 105 proteins.

(**e**, **f**) Relative protein abundance represented as normalized spectra abundance factor (NSAF) values clustered using Pearson correlation as a distance metric and Ward as a method. Each column represents an individual purification and each row represents a prey protein. The color intensity depicts the protein abundance with the brightest yellow indicating highest abundance and lower intensity indicating decreasing abundance. Black indicates that the protein was not detected in a particular sample.

(**e**) Hierarchical clustering of the 105 proteins identified with less stringent criteria.

(**f**) Hierarchical clustering of the 22 proteins identified with a more stringent method using the QSpec software.

Rere protein clustered with Wdr5, Hdac1 and Hdac2 proteins with the two different methods used for analysis.

Supplementary Figure 2

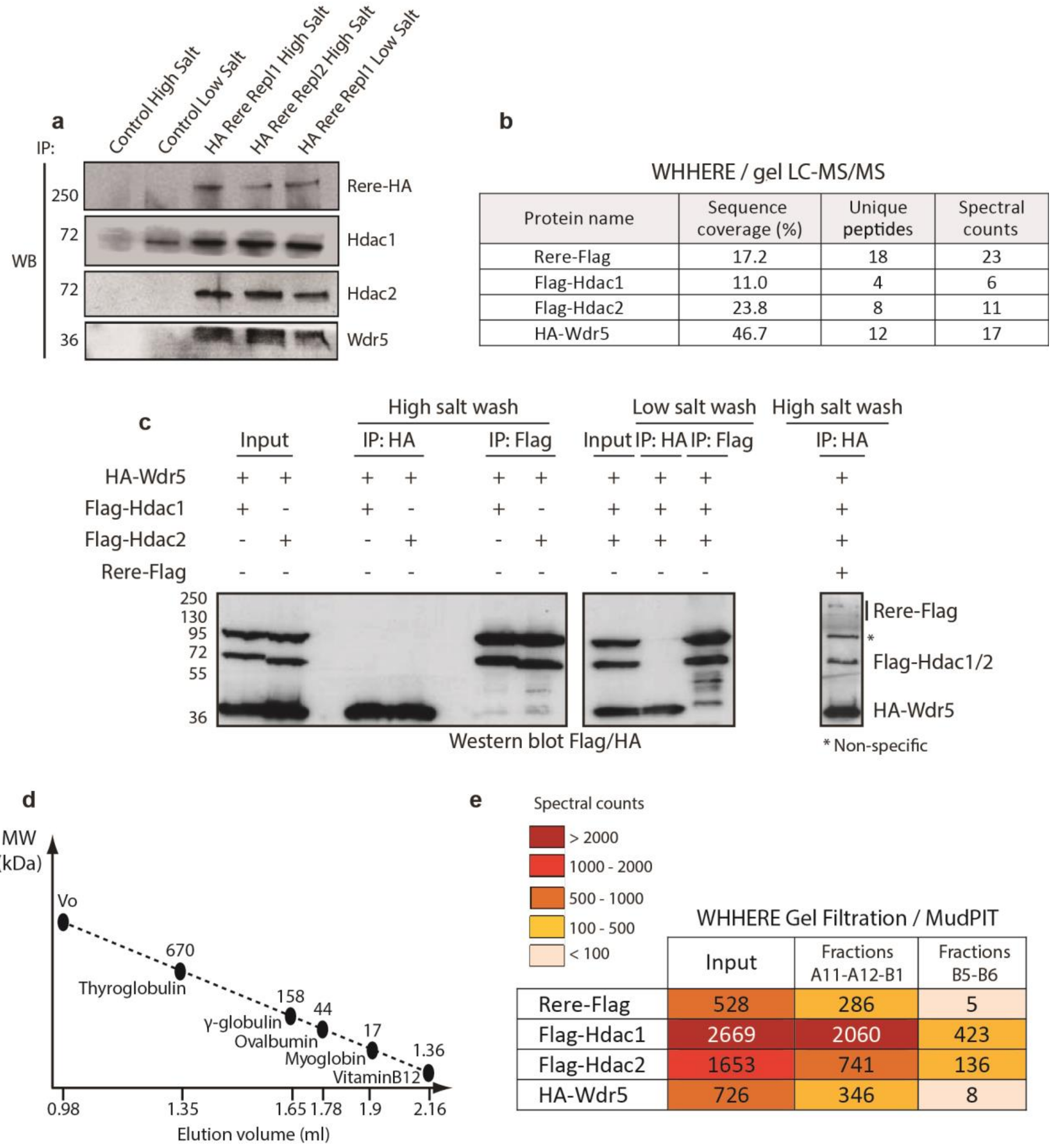

Protein name	Sequence coverage (%)	Unique peptides	Spectral counts
Rere-Flag	17.2	18	23
Flag-Hdac1	11.0	4	6
Flag-Hdac2	23.8	8	11
HA-Wdr5	46.7	12	17

	Input	Fractions A11-A12-B1	Fractions B5-B6
Rere-Flag	528	286	5
Flag-Hdac1	2669	2060	423
Flag-Hdac2	1653	741	136
HA-Wdr5	726	346	8

Supplementary Figure 2 (related to Figure 1) Biochemical characterization of the WHHERE complex.

(**a**) Western blot analysis of Rere-HA immunoprecipitation eluates from *T-Cre;RS-Rere-HA* mouse embryos using antibodies against HA, Hdac1, Hdac2 and Wdr5. Replicate 1 (Repl1) and Replicate 2 (Repl2).

(**b**) Protein sequence coverage (%), number of unique peptides and spectral counts obtained from the gel LC-MS/MS analysis of Coomassie stained gel bands (shown in Fig. 1c) containing the recombinant WHHERE complex members (Rere-Flag, Flag-Hdac1, Flag-Hdac2 and HA-Wdr5).

(**c**) Flag or HA immunoprecipitation from extracts of cells infected with baculoviruses expressing HA-Wdr5, Flag-Hdac1, Flag-Hdac2 and Rere-Flag at low (150 mM KCl) and/or high (500 mM KCl) salt wash. Flag and HA western blot.

(**d**) Standard curve performed on the gel filtration column to define the molecular weight (MW) of the WHHERE complex. The MW of Thyroglobulin (670 kDa), ɣ-globulin (158 kDa), Ovalbumin (44 kDa), Myoglobulin (17 kDa) and Vitamin B12 (1355 Da) was plotted based on their elution volume on the Superose 6 column in which the WHHERE complex was analyzed.

(**e**) MudPIT analysis of the recombinant WHHERE complex before gel filtration analysis (Input) and after gel filtration from pooled elution fractions containing (A11-A12-B1) or not (B5-B6) the WHHERE complex.

Supplementary Figure 3

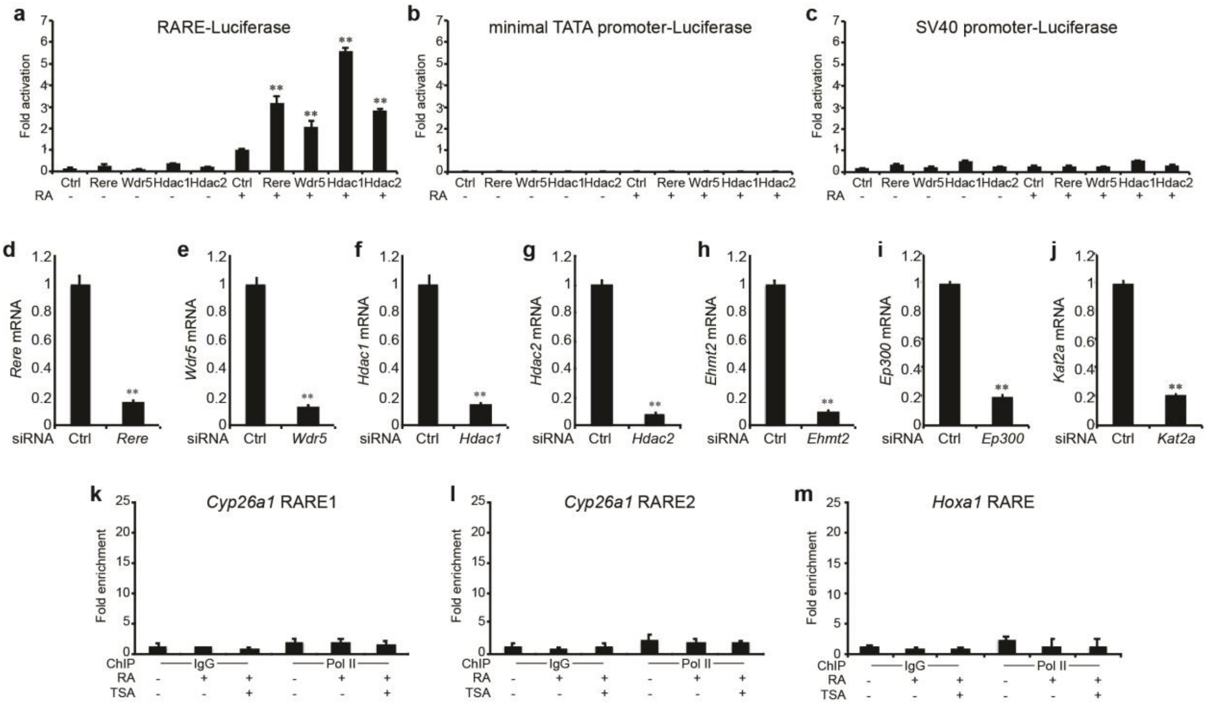

Supplementary Figure 3 (related to Figures 2, 4, 5 and 7) WHHERE complex specifically activates the RARE-Luciferase reporter.

(**a**-**c**) RARE-Luciferase (**a**), minimal TATA promoter-Luciferase (**b**) and SV40 promoter-Luciferase (**c**) activity from NIH3T3 cells treated or not with 1 µM RA for 20 hours. Cells transfected with expression plasmids containing *Rere*, *Wdr5*, *Hdac1* or *Hdac2* (n = 3).

(**d**-**j**) Transfection of NIH3T3 cells with siRNAs targeting *Rere* (**d**), *Wdr5* (**e**), *Hdac1* (**f**), *Hdac2* (**g**), *Ehmt2* (**h**), *Ep300* (**i**) and *Kat2a* (**j**). qPCR analysis of *Rere* (**d**), *Wdr5* (**e**), *Hdac1* (**f**), *Hdac2* (**g**), *Ehmt2* (**h**), *Ep300* (**i**) and *Kat2a* (**j**) mRNA. Expression level of each gene was normalized to *Rplp0* mRNA (n = 4).

(**k**-**m**) Pol II occupancy on the *Cyp26a1* RARE1, *Cyp26a1* RARE2 and *Hoxa1* RARE elements. ChIP analysis of the *Cyp26a1* RARE1 (**k**), *Cyp26a1* RARE2 (**l**) and *Hoxa1* RARE (**m**) elements with Pol II antibody using NIH3T3 cells treated with 1 µM RA or 1 µM RA and 100 nM TSA for 1 hour (data represent mean ± s.d. from triplicate PCR reactions).

In all graphs data represent mean ± s.e.m. unless otherwise specified. $^{**}P < 0.01$.

Supplementary Figure 4

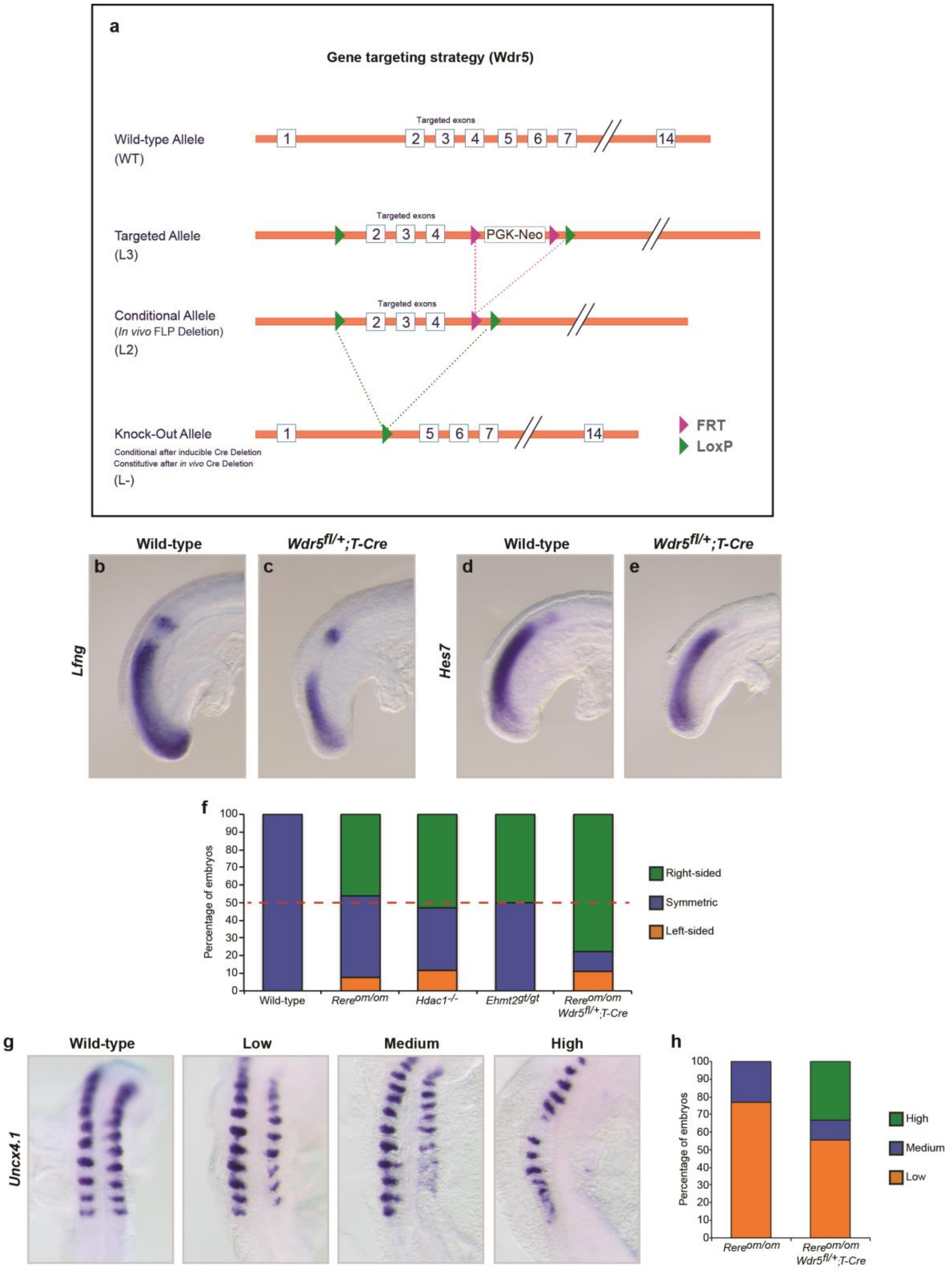

Supplementary Figure 4 (related to Figures 3 and 5) Asymmetric somite development in mutant embryos for *Rere, Hdac1*, *Wdr5* and *Ehmt2*.

(**a**) *Wdr5* conditional allele (*Wdr5*$^{fl/+}$) mice were generated by inserting two loxP sites flanking the region encompassing exon 2 to exon 4 in C57BL/6N-derived ES cells. The Neo selection cassette flanked by FRT site was removed in vivo using a FlpO deleter mice. The removal of exon 2, 3 and 4 with a Cre deleter mice led to early embryonic lethality before E8.5.

(**b-e**) Normal expression of Notch-related genes, *Lfng* and *Hes7*, in *Wdr5*$^{fl/+}$*;T-Cre* embryos. *In situ* hybridization for *Lfng* (**b** and **c**) and *Hes7* (**d** and **e**) in wild-type (**b** and **d**) and *Wdr5*$^{fl/+}$*;T-Cre* (**c** and **e**) embryos at E9.0-E9.5 (lateral views).

(**f**) Graph representing the percentage of 7- to15-somite stage embryos with left-sided (orange), symmetric (blue) or right-sided (green) delay in somite formation in wild-type, *Rere*$^{om/om}$, *Hdac1*$^{-/-}$, *Ehmt2*$^{gt/gt}$ and *Rere*$^{om/om}$*;Wdr5*$^{fl/+}$*;T-Cre* embryos.

(**g**) *In situ* hybridization for *Uncx4.1* in wild-type and mutant embryos representing different categories of phenotype severity: Low, Medium and High.

(**h**) Graph representing the percentage of embryos with Low, Medium and High right-sided phenotype severity in *Rere*$^{om/om}$ and *Rere*$^{om/om}$*;Wdr5*$^{fl/+}$*;T-Cre* embryos.

Supplementary Figure 5

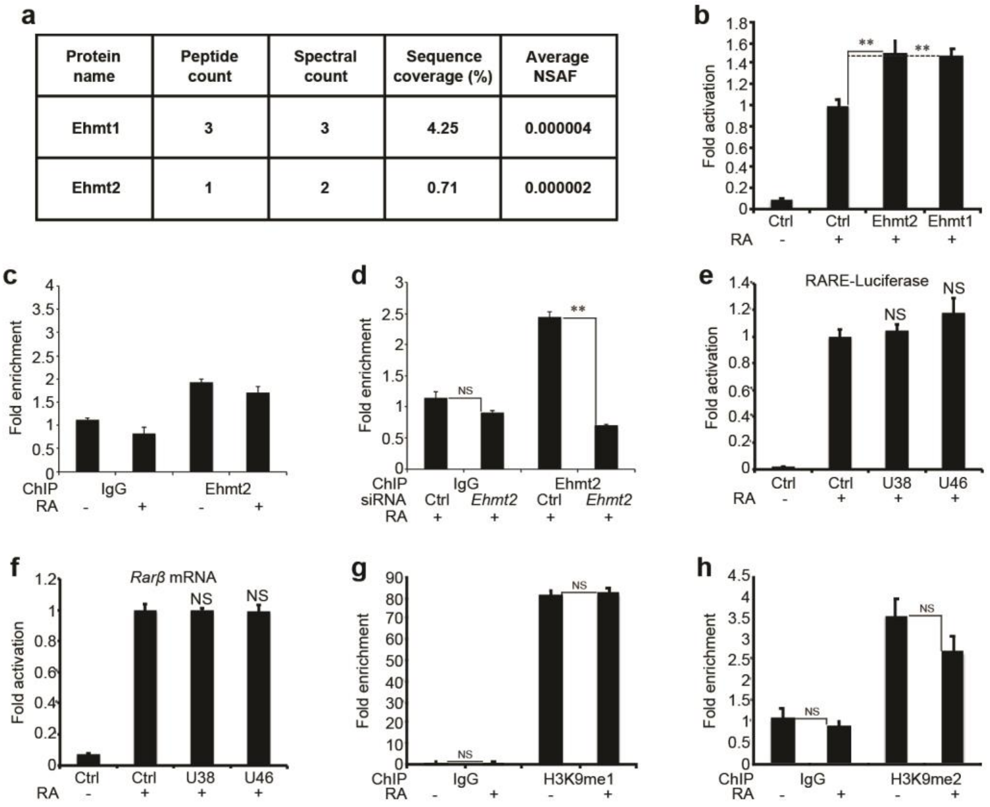

Protein name	Peptide count	Spectral count	Sequence coverage (%)	Average NSAF
Ehmt1	3	3	4.25	0.000004
Ehmt2	1	2	0.71	0.000002

Supplementary Figure 5 (related to Figure 5) Ehmt2 and the family-related protein Ehmt1 regulate retinoic acid signalling.

(**a**) Table representing the peptide count, spectral count, percentage of coverage (%) and average NSAF values for Ehmt1 and Ehmt2 from the MUDPIT analysis of the in vivo Rere-HA immunoprecipitation.

(**b**) RARE-Luciferase activity from NIH3T3 cells treated or not with 1 µM RA for 20 hours. Cells were transfected with one of the expression plasmids containing *Ehmt2* or *Ehmt1* (n = 4).

(**c**) ChIP analysis of an upstream region (-3 Kb) of the *Rarβ* promoter with a specific antibody for Ehmt2 using NIH3T3 cells treated or not with 1 µM RA during 1 hour (data represent mean ± s.d. from triplicate PCR reactions).

(**d**) ChIP analysis of the RARE element in the *Rarβ* promoter using NIH3T3 cells transfected with siRNA for *Ehmt2* and treated with 1 µM RA during 1 hour. ChIP was performed with an antibody specific to Ehmt2 (n = 3).

(**e**) RARE-Luciferase activity from NIH3T3 cells treated or not with 1 µM RA for 20 hours and with the Ehmt2 methyltransferase inhibitors UNC0638 (U38) (2µM) or UNC0646 (U46) (2µM) (n = 4).

(**f**) *Rarβ* mRNA expression from NIH3T3 cells treated or not with 1 µM RA for 20 hours and with the Ehmt2 methyltransferase inhibitors UNC0638 (U38) (2µM) or UNC0646 (U46) (2µM) (n = 4).

(**g**, **h**) ChIP analysis of the *Rarβ* promoter from NIH3T3 cells treated with 1 µM RA during 1 hour. ChIP was performed with antibodies specific to H3K9me1 (**e**) and H3K9me2 (**f**) (n = 3).

In all graphs data represent mean ± s.e.m. unless otherwise specified. NS – not significant, *$P < 0.05$ and **$P < 0.01$.

Supplementary Figure 6

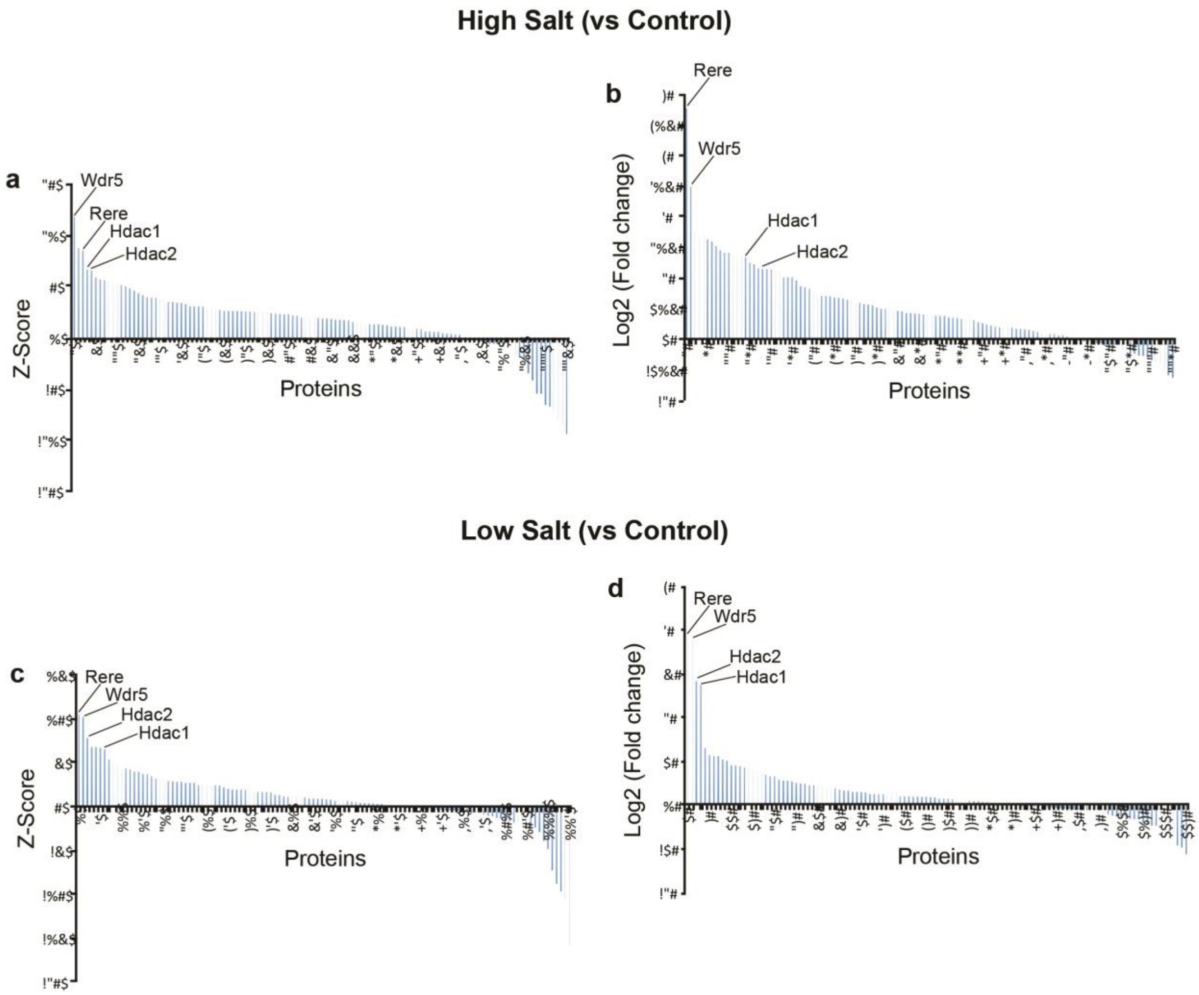

Supplementary Figure 6: Statistical analysis of the proteomics data using QSpec software. (related to Figure 1)

(**a**, **b**) Graphs representing the Z-Score (**a**) and Log2 (Fold change) (**b**) analysis of the Rere-HA immunopurifications from high salt conditions.

(**c**, **d**) Graphs showing the Z-Score (**c**) and Log2 (Fold change) (**d**) analysis of the Rere-HA immunoprecipitations from low salt conditions.

Rere, Wdr5, Hdac1 and Hdac2 are among the proteins with the highest Z-Score and Log2 (Fold change) in the different immunopurification conditions supporting the formation of a complex containing the four proteins.

Supplementary Figure 7

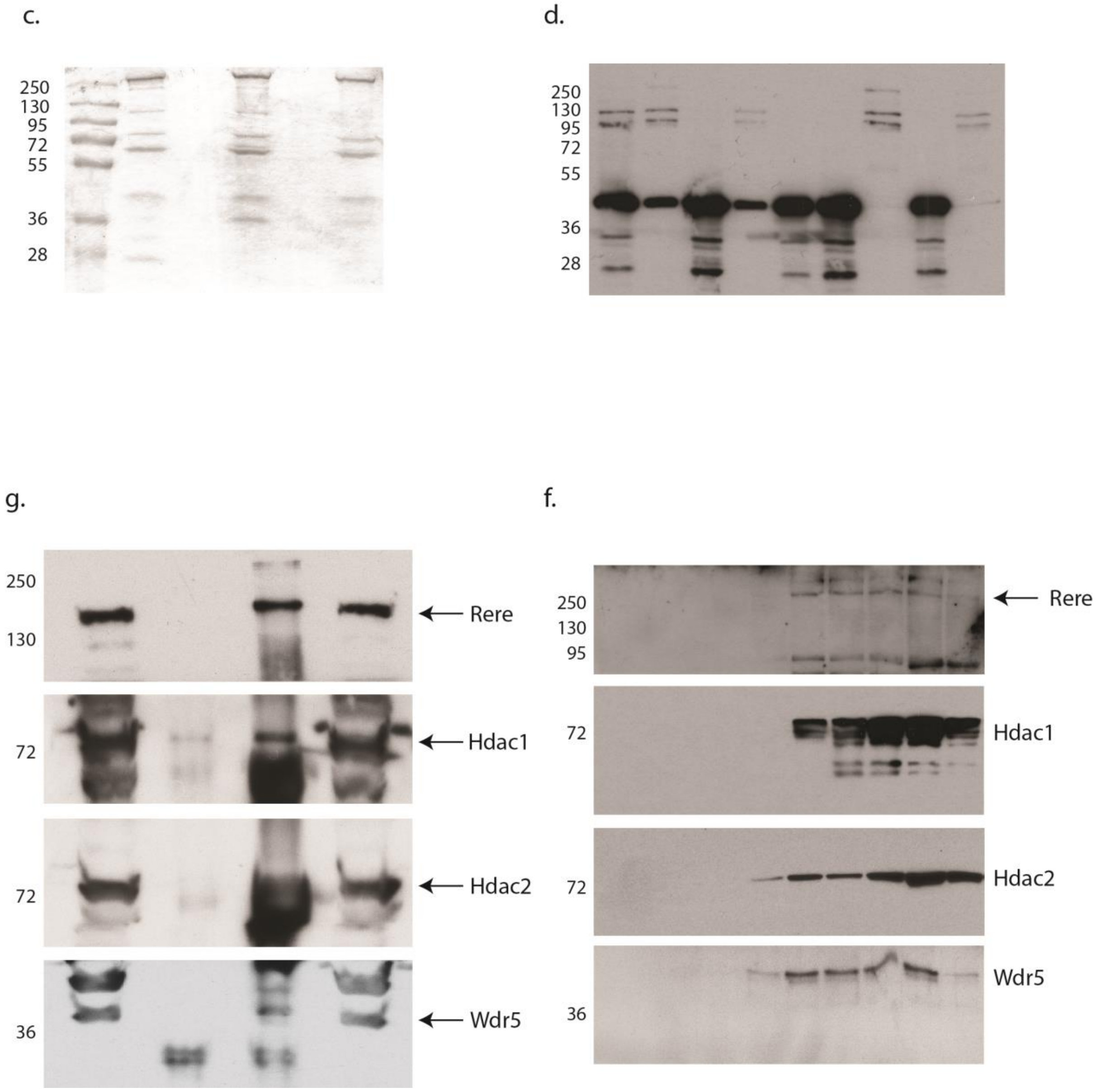

Supplementary Figure 7: Uncropped blots shown in Figure 1 (related to Figure 1). Panels are labeled as in the related Figure.

Supplementary Figure 8

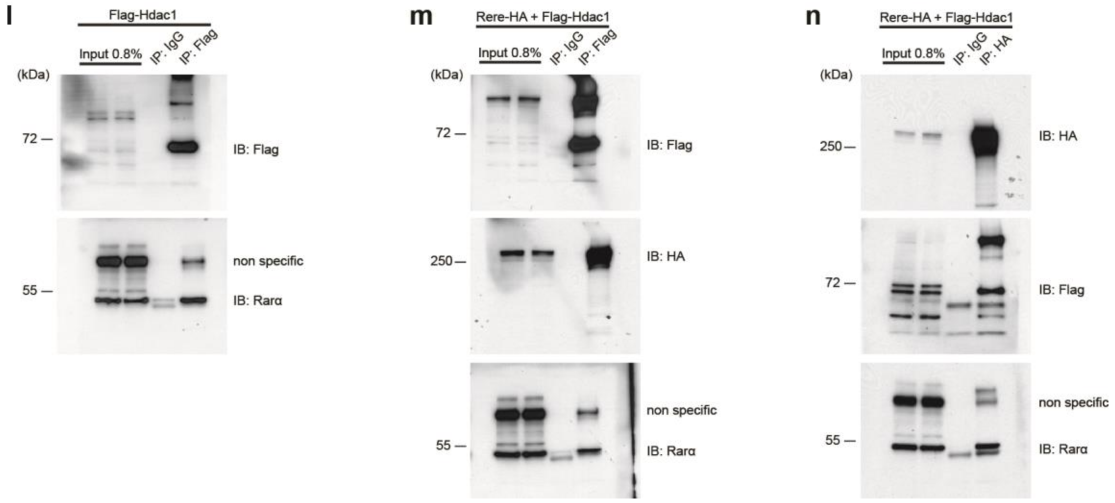

Supplementary Figure 8: Uncropped blots shown in Figure 4 (related to Figure 4). Panels are labeled as in the related Figure.

Supplementary Figure 9

a.

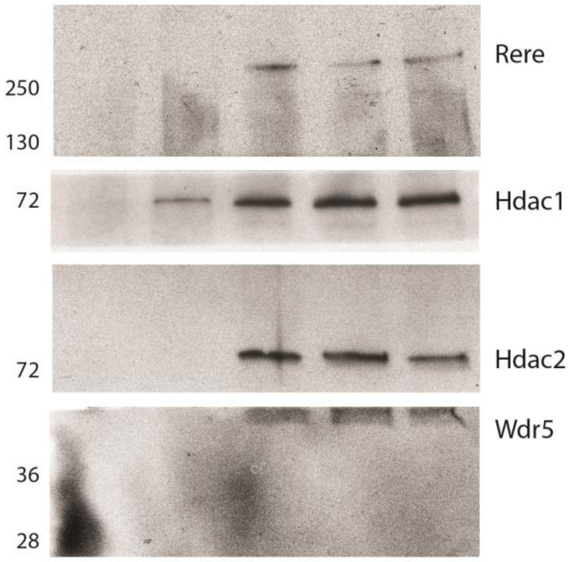

c.

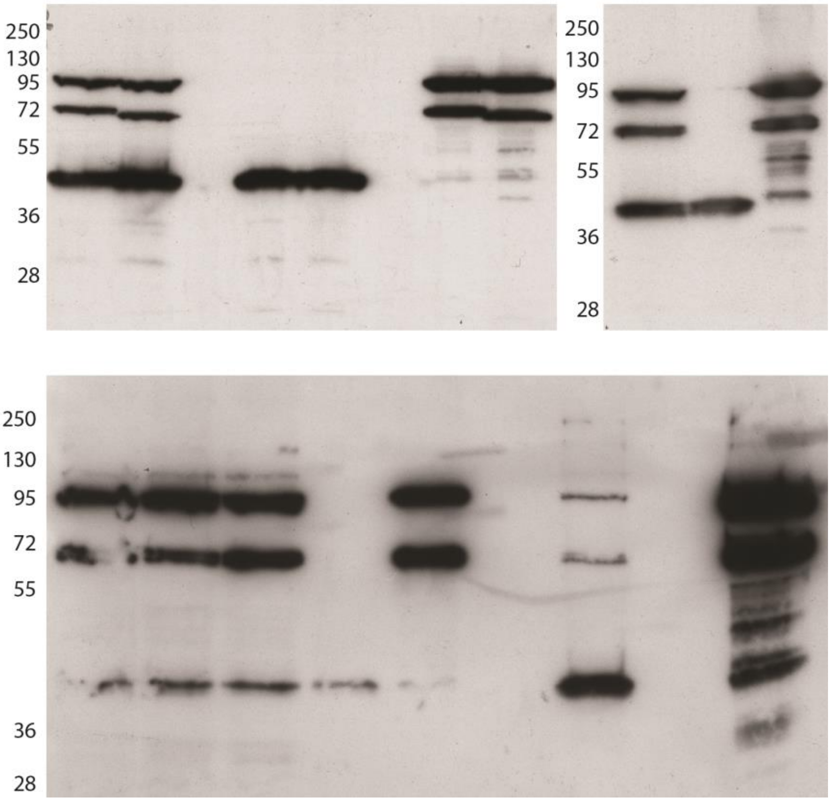

Supplementary Figure 9: Uncropped blots shown in Supplementary Figure 2 (related to Supplementary Figure 2). Panels are labeled as in the related Figure.

Supplementary Table 1: List of primers used for ChIP experiments.

Gene	Primer sequence
Rarβ (RARE)	For: TTAAGCGCTGTGAGAATCCTG
	Rev: ACCTTCAAATGACCCAACCA
Rarβ (-3 Kb)	For: ACAACCACAAAGAGCCTTGC
	Rev: GCAAGGGGACATAGCTGAAA
RARE-LacZ	For: CCACCAATCCCCATATGGAA
	Rev: TGCTGCACGCGGAAGA
Cyp26a1 (RARE1)	For: CCCGATCCGCAATTAAAGATGA
	Rev: CTTTATAAGGCCGCCCAGGTTAC
Cyp26a1 (RARE2)	For: TTCACTGAGATGTCACGGTCC
	Rev: TTCCCAATCCTTTAGCCTGA
Hoxa1 (RARE)	For: TCTTGCTGTGACTGTGAAGTCG
	Rev: GAGCTCAGATAAACTGCTGGGACT

Supplementary Note 1

The following workflow was used for the identification of proteins associated to Rere through large-scale affinity purification and mass spectrometry:

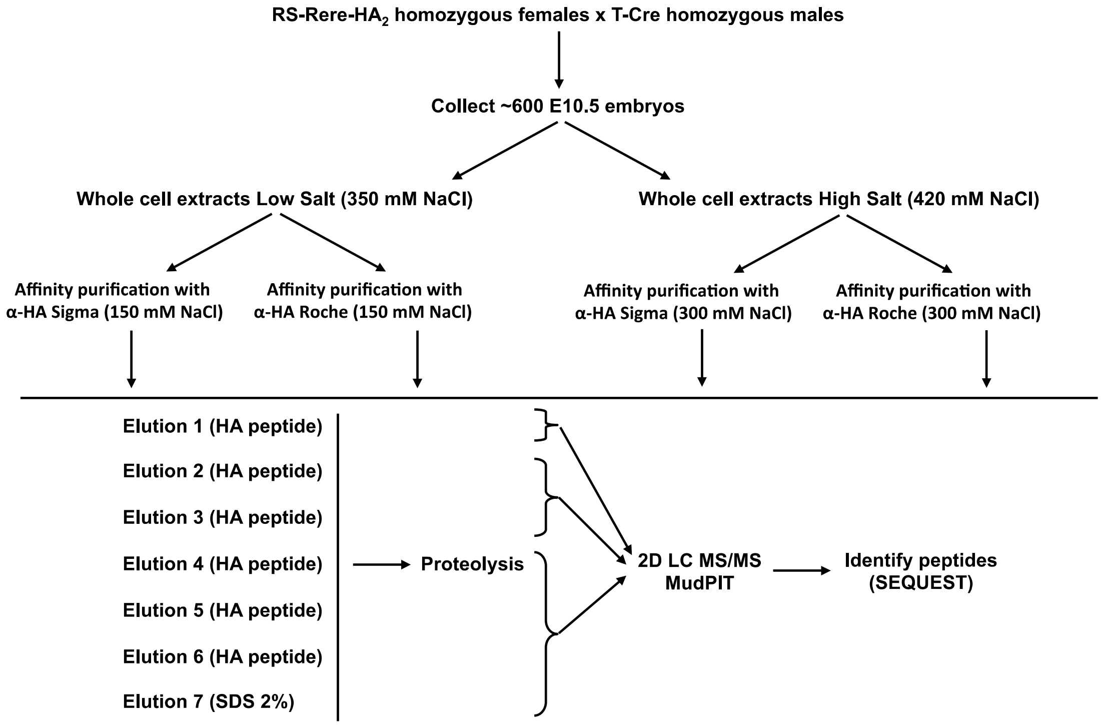

A total of 23 different immunoprecipitations from *RS-Rere-HA* embryos and 12 immunoprecipitations from wild-type control embryos were performed and submitted to MudPIT analysis. This led to the identification of a total of 3197 proteins. For each purification, a protein was considered to be specific for the bait if its NSAF value was greater than its corresponding control. The nonspecific proteins were replaced with 0 in the dataset. This resulted in a list of 2713 identified proteins. To reduce the number of hits we only kept proteins present in at least two replicates in the low salt samples and at least two times in the high salt samples. The obtained 105 proteins (the 116 entries common to all conditions corresponds to 105 non-duplicated proteins) were next subject to clustering analysis to determine proteins showing the closest distribution to the bait protein - Rere (Supplementary

Fig. 1d). Rere was found in a cluster together with Wdr5, Hdac1 and Hdac2 (Supplementary Fig. 1e). To confirm these interactions using a different statistical tool we applied a more stringent criteria to the 105 identified proteins. Statistical analysis based on the QSpec software (http://www.nesvilab.org/qspec.php/)[64] was performed on the proteomics data to detect differential protein expression between the controls and the different immunopurification conditions (i.e. low and high salt). QSpec calculates the ratio of likelihood for differential expression for each protein based on certain model assumptions (Poisson-family distributions for count data) and a Z-statistics score. High confidence interactions have a Z-score value of ≥ +2. A total of 22 proteins showed a +2 or higher Z-score in analyses comparing controls versus immunopurifications in high salt conditions and controls versus immunopurifications in low salt conditions (Supplementary Fig. 1f). Among the highest Z-score values we can find Rere, Wdr5, Hdac1 and Hdac2 confirming the high confidence associations between these proteins (Supplementary Figure 6).

Supplementary References

1. Choi, H., Fermin, D. & Nesvizhskii, A.I. Significance analysis of spectral count data in label-free shotgun proteomics. *Mol Cell Proteomics* **7**, 2373-2385 (2008).
